# An Injection Leading to Oesophageal Perforation: A Rare Case of Contained Boerhaave’s Syndrome Following Unsupervised GLP-1/GIP Receptor Agonist Dose Escalation

**DOI:** 10.7759/cureus.101811

**Published:** 2026-01-18

**Authors:** Noor Sadiq Syed, Thomas Oldfield, Syed Akash-Ul-Husnain, Atif Moiz

**Affiliations:** 1 Emergency Medicine, Mid Yorkshire Teaching NHS Trust, Wakefield, GBR

**Keywords:** adolescent nutrition, community medicine & public health, general pharmacology, special interest in emergency medicine, weight loss and obesity

## Abstract

Oesophageal perforation, also known as Boerhaave’s syndrome, is a rare but potentially fatal condition that classically results from forceful emesis. Prompt diagnosis is critical, as delays are associated with a significant increase in morbidity and mortality. We present the case of an 18-year-old woman who developed severe and persistent vomiting after self-escalation of a dual glucose-dependent insulinotropic polypeptide (GIP) and glucagon-like peptide-1 (GLP-1) receptor agonist prescribed for weight loss, culminating in a contained oesophageal perforation. Imaging demonstrated a pneumothorax and extensive mediastinal emphysema (pneumomediastinum), without evidence of ongoing contrast extravasation on water-soluble swallow, suggesting spontaneous sealing of the perforation. The patient was successfully managed conservatively on a medical ward with intravenous (IV) antibiotics, IV fluids, proton pump inhibitor (PPI) therapy, and close clinical observation.

This case highlights the importance of recognising severe vomiting associated with GLP-1/GIP receptor agonist therapy as a potential precipitant of oesophageal injury and demonstrates that carefully selected contained perforations may be successfully managed conservatively with close multidisciplinary monitoring.

## Introduction

Boerhaave’s syndrome is a spontaneous full-thickness rupture of the oesophagus caused by a sudden rise in intra-oesophageal pressure, most commonly due to forceful retching or vomiting. Although rare, with an estimated incidence of approximately 3.1 cases per million per year, it is associated with a high mortality rate of 20%-40%, particularly when diagnosis is delayed beyond 24 hours [1,2]. The classic Mackler triad (vomiting, chest pain, and subcutaneous emphysema) is observed in fewer than half of cases, making early diagnosis challenging.

Glucagon-like peptide-1 (GLP-1) receptor agonists and newer dual glucose-dependent insulinotropic polypeptide (GIP)/GLP-1 receptor agonist therapies are increasingly used for weight management. Their mechanism of action includes delayed gastric emptying, appetite suppression, and glycaemic modulation. Gastrointestinal adverse effects, such as nausea and vomiting, are well recognised, particularly during dose-escalation phases [3-5]. While such symptoms are typically self-limiting, severe complications, including oesophageal injury, may occur. We report a case of Boerhaave’s syndrome in an adolescent patient following unsupervised escalation of a dual GIP/GLP-1 receptor agonist, highlighting the importance of safe medication titration and the viability of conservative management in appropriately selected cases. GLP-1 receptor agonists and dual GIP/GLP-1 receptor agonists exert their weight-loss effects primarily through appetite suppression and delayed gastric emptying. This delay can lead to increased intragastric volume and pressure, particularly during dose-escalation phases. In the setting of repeated forceful vomiting, a sudden rise in intra-oesophageal pressure against a closed glottis may occur, predisposing to full-thickness oesophageal rupture, the underlying mechanism of Boerhaave’s syndrome.

## Case presentation

An 18-year-old woman with no significant past medical history presented to the Emergency Department with a four-day history of severe, persistent vomiting, associated with pleuritic chest pain. Her symptoms began shortly after she independently increased the dose of her prescribed dual GIP and GLP-1 receptor agonist injection, which had been initiated for weight loss. She reported repeated episodes of forceful vomiting, initially producing dark brown material, and later progressing to greenish, bilious fluid. She denied any episodes of fresh hematemesis, dysphagia, odynophagia, or melena.

On arrival, she was tachycardic, with a heart rate of 114 beats per minute, but otherwise haemodynamically stable, with a blood pressure of 128/86 mmHg, a temperature of 37.1°C, and an oxygen saturation of 96% on room air. Physical examination revealed mild epigastric tenderness, without guarding or rigidity. There was no palpable subcutaneous emphysema in the neck or chest wall, and cardiopulmonary examination was otherwise unremarkable.

Initial laboratory investigations demonstrated a raised white cell count of 18.9 × 10⁹/L (reference range: 4-11 × 10⁹/L) and an elevated C-reactive protein (CRP) of 17 mg/L (reference range: <5 mg/L), which was suggestive of an inflammatory response. Electrolytes, renal function, and liver function tests were within normal limits, with reference ranges detailed in the manuscript.

**Figure 1:**
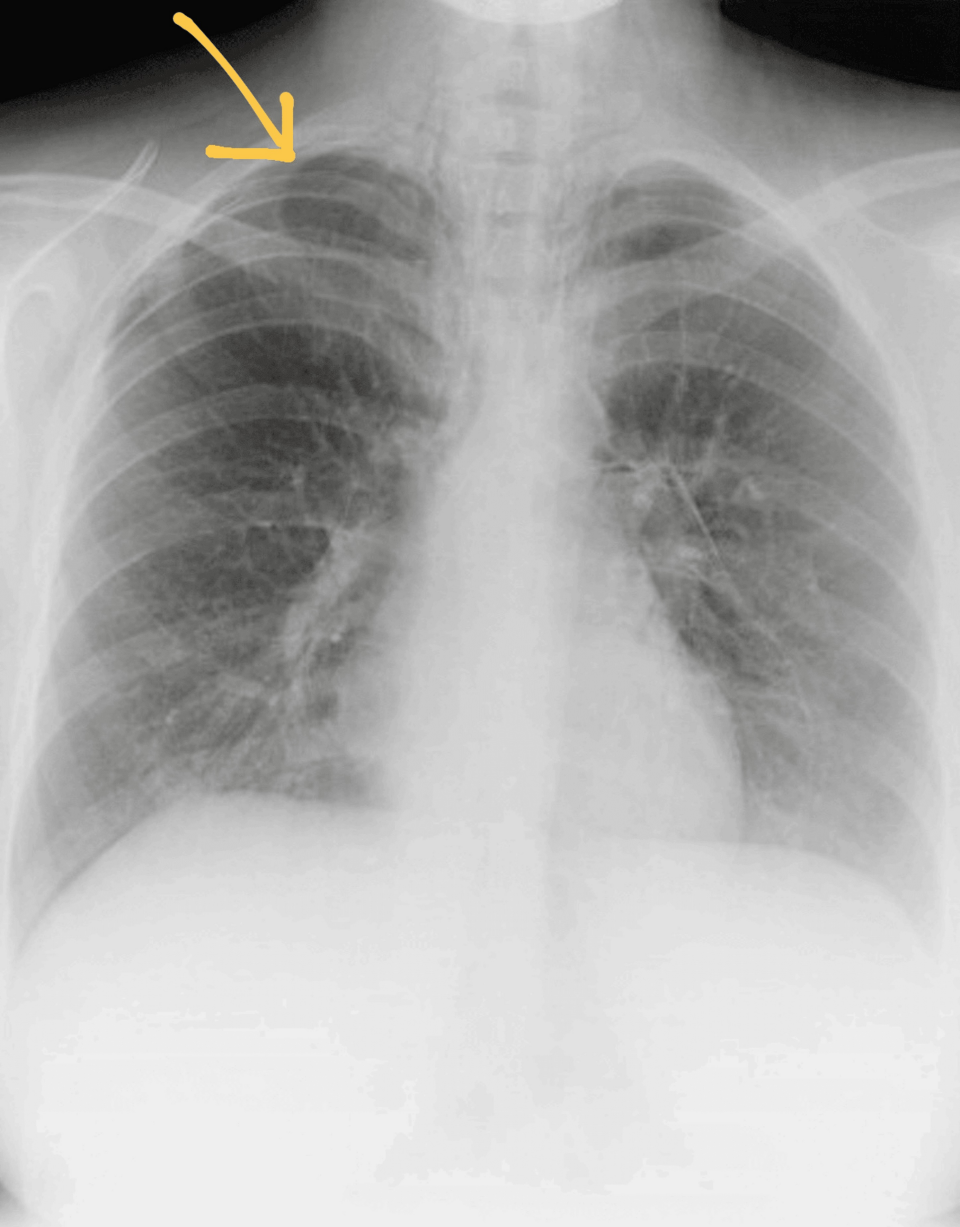
Chest radiograph showing right-sided pneumothorax with mediastinal air.

A chest radiograph revealed a small, right-sided pneumothorax with associated mediastinal air (Figure 1).

**Figure 2:**
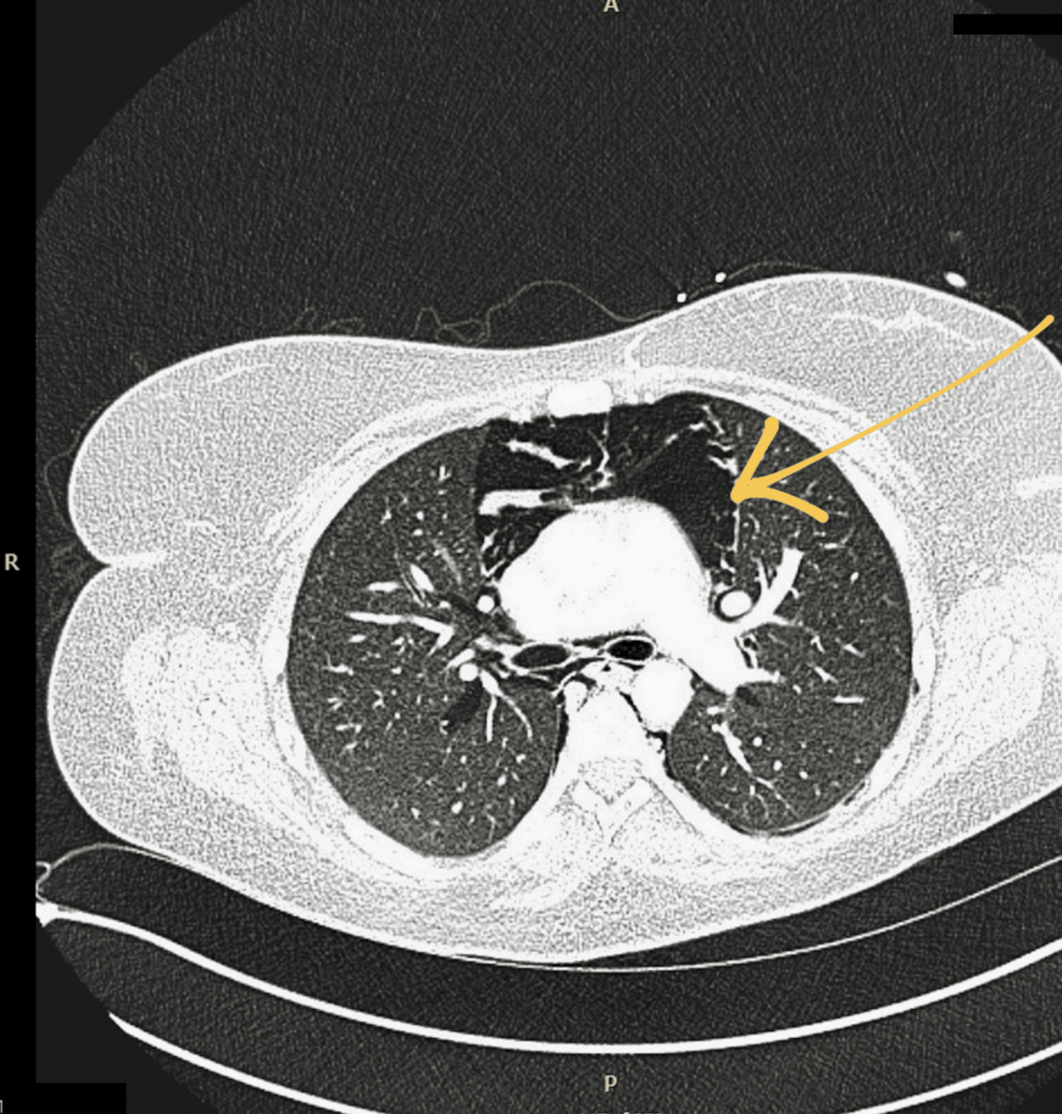
Axial CT image demonstrating pneumomediastinum and air tracking into the neck soft tissues. CT, computed tomography

Given the concerning clinical context and radiographic findings, a computed tomography (CT) scan of the thorax was performed, which confirmed extensive pneumomediastinum, with air tracking into the cervical fascial planes. No associated fluid collections, abscesses, or pleural effusions were identified (Figure 2).

**Figure 3:**
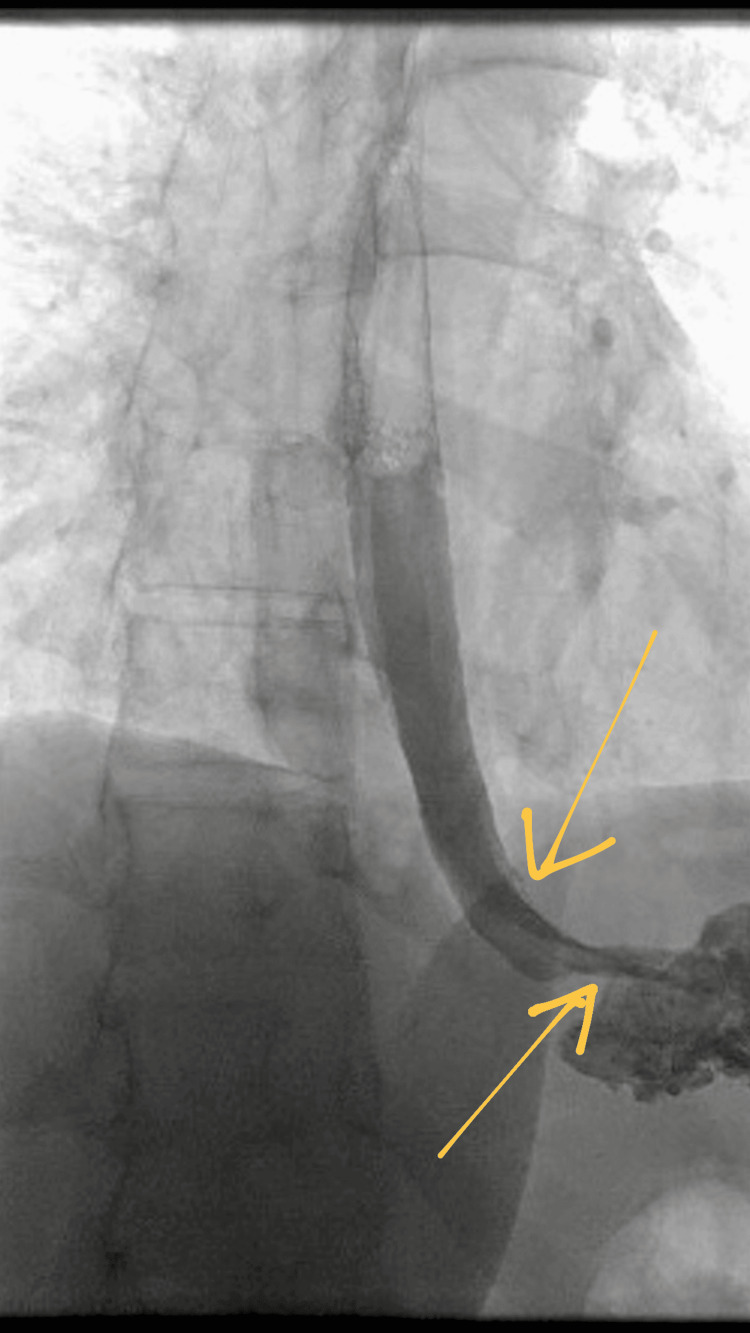
Water-soluble contrast swallow study showing no contrast extravasation.

To further evaluate for oesophageal perforation, a water-soluble contrast swallow study was undertaken. This demonstrated no contrast extravasation, suggesting either the absence of a perforation or a small, contained oesophageal injury (Figure 3).

In view of her clinical stability, lack of overt sepsis, and negative contrast study, the cardiothoracic surgery team recommended conservative management.

The patient was managed with bowel rest (nil by mouth), intravenous (IV) fluid resuscitation, IV piperacillin-tazobactam, and proton pump inhibitor (PPI) therapy, alongside close clinical observation and serial examinations. Over the subsequent five days, her chest pain and vomiting resolved, and inflammatory markers progressively normalised. Oral intake was cautiously reintroduced without complication, and she was discharged home on day 7 of admission. At outpatient follow-up, she remained asymptomatic, with no recurrence of symptoms.

She had no significant past medical history, took no regular medications other than the prescribed weight-loss injection, and reported no alcohol misuse, eating disorder behaviours, caustic ingestion, or prior oesophageal disease.

## Discussion

Boerhaave’s syndrome occurs when a sudden rise in intra-oesophageal pressure against a closed glottis leads to full-thickness rupture, most commonly in the distal posterolateral oesophagus. Delayed gastric emptying and repeated vomiting significantly increase the risk of this mechanism. The delayed gastric emptying associated with GLP-1/GIP receptor agonists increases intragastric pressure, which, when combined with repeated forceful emesis, can result in a sudden rise in intra-oesophageal pressure, sufficient to cause transmural rupture. This pharmacologically mediated mechanism is particularly relevant during unsupervised or rapid dose escalation.

Dual GIP/GLP-1 receptor agonists - including tirzepatide-based therapies - are increasingly used for weight loss. Their mechanism involves delayed gastric emptying, appetite suppression, and improved glycaemic control. Gastrointestinal side effects, such as nausea and vomiting, are common, particularly during early treatment or dose escalation. These criteria are consistent with the selective nonoperative approach described by Cameron et al. [3,4].

This case is clinically significant due to several distinguishing features. The patient was unusually young, had no underlying comorbidities, and developed Boerhaave’s syndrome following unsupervised escalation of GLP-1/GIP receptor agonist therapy. Notably, she did not present with the full Mackler triad, and imaging demonstrated a contained, self-sealing perforation, without pleural contamination or systemic sepsis. Successful conservative management in this context highlights both the severity of vomiting-induced oesophageal injury and the feasibility of non-operative treatment in carefully selected, clinically stable patients. To our knowledge, such presentations remain under-reported in the setting of modern weight-loss pharmacotherapy.

In this case, the patient’s unsupervised dose increase precipitated sustained vomiting, ultimately leading to a contained oesophageal tear. While surgical repair is the gold standard for most oesophageal perforations, nonoperative management may be considered when the perforation is contained, there is no evidence of systemic sepsis, no pleural contamination is present, and close multidisciplinary monitoring is available.

CT imaging remains the most useful modality for assessing pneumomediastinum, pleural involvement, and complications, while contrast swallow studies help evaluate ongoing leaks [6,7].

This case also underscores the need for robust patient counselling regarding medication titration, expectations during escalation phases, and potential gastrointestinal risks.

## Conclusions

This case highlights a rare but clinically important complication of severe vomiting following unsupervised escalation of injectable weight-loss therapy. Clinicians should maintain a high index of suspicion for Boerhaave’s syndrome in patients presenting with chest pain after vomiting, particularly when medications that delay gastric emptying are involved. Importantly, conservative management may be appropriate in highly selected patients with contained oesophageal perforations, who remain clinically stable and are managed with close multidisciplinary oversight.
